# Vascularized fibular grafting following tumor resection demonstrates acceptable long-term outcomes in Denmark: a national retrospective cohort study

**DOI:** 10.2340/17453674.2025.42848

**Published:** 2025-01-13

**Authors:** Christian Lind NIELSEN, Daniel Thor Halberg DYBDAL, Peter VESTER-GLOWINSKI, Lisa Lyngsie HJALGRIM, Pernille Edslev WENDTLAND, Birgitte Jul KIIL, Michael Melchior BENDTSEN, Michael Mørk PETERSEN, Thomas BAAD-HANSEN

**Affiliations:** 1 Department of Orthopedic Surgery, Aarhus University Hospital, Aarhus; 2 Department of Paediatrics and Adolescent Medicine, Rigshospitalet, University of Copenhagen, Copenhagen; 3 Department of Plastic Surgery and Burns Treatment, Rigshospitalet, University of Copenhagen, Copenhagen; 4 Department of Paediatrics and Adolescent Medicine, Aarhus University Hospital, Aarhus; 5 Department of Plastic and Breast Surgery, Aarhus University Hospital, Aarhus; 6 Department of Orthopedic Surgery, Rigshospitalet, University of Copenhagen, Copenhagen, Denmark

## Abstract

**Background and purpose:**

Vascularized fibular grafting following tumor resection is an essential treatment option in limb salvage surgery. We aimed to evaluate: (I) bone healing, (II) complications and reoperations, (III) limb salvage, and (IV) survival.

**Methods:**

We present a retrospective evaluation of a national cohort comprising 27 patients. The indications were 13 cases of Ewing sarcoma, 12 cases of osteosarcoma, and 2 cases of giant cell tumor. The median age at surgery was 16 years (interquartile range [IQR] 10–18), and the median follow-up was 82 months (IQR 32–101). Patients were analyzed overall, as well as in subgroups based on tumor location (upper versus lower extremity) and pathology (osteosarcoma versus Ewing sarcoma).

**Results:**

The primary rate of graft union was 63%, and after secondary procedures the overall rate of graft union was 67%, with a median time to union of 13 months (IQR 9–17). The reoperation rate was 74%, while the limb salvage rate was 93%. The 5-year overall survival rate was 81% (95% confidence interval [CI] 61–92). Patients with upper extremity tumors were more likely to attain graft union (risk ratio [RR] 5.5, CI 1.3–31.5) and less likely to undergo multiple reoperations (RR 0.3, CI 0.8–0.9) than patients with lower extremity tumors.

**Conclusion:**

Vascularized fibular grafting following tumor resection was associated with a graft union rate of 67%, a high frequency of reoperations, a high limb salvage rate (93%), and a 5-year survival rate of 81%.

Bone sarcomas are rare malignancies that primarily affect children and adolescents [1]. Although only 4,000 new cases of bone sarcomas are diagnosed annually in the United States, they remain the third most common cause of cancer-related deaths in individuals under 19 years [2]. Historically, amputations were the primary treatment for bone sarcomas in the extremities. However, advancements in systemic chemotherapy protocols have made limb salvage surgery a viable alternative. Currently, limb salvage surgery is feasible in 90–95% of extremity bone sarcomas cases, with a 5-year survival rate of 70–80% in non-metastatic disease [3,4].

Reconstruction using expandable endoprostheses is the preferred method following periarticular tumor resection in the lower extremities. However, complications such as deep infection, aseptic loosening, and implant failure are frequent [5-7].

Vascularized fibular grafting (VFG) was first introduced in orthopedic oncology in 1977 and has since been utilized for the reconstruction of bone defects caused by trauma, infection, or congenital anomalies [8-10]. VFG is considered the gold standard and workhorse in biological reconstruction due to its size, shape, and intact blood supply, which promotes early hypertrophy and the delivery of high local concentrations of antibiotics [11-14]. VFG is indicated in diaphyseal or intraarticular defects in the upper extremity [15]. However, a high rate of postoperative complications has been reported, the most common being nonunion, fractures, and infections, which necessitate additional surgeries [16-19]. Consequently, evaluating patient outcomes is crucial for optimizing care and tailoring treatment strategies. Despite its widespread adoption, no prior studies have provided a comprehensive evaluation of all patients treated within a single country.

We aimed to evaluate a national cohort of 27 patients who underwent VFG following tumor resection in Denmark between 2010 and 2022. 4 key surgical and oncological parameters were evaluated: (I) bone healing, (II) complications and reoperations, (III) limb salvage, and (IV) survival.

## Methods

### Study design

In this national retrospective cohort study, we systematically reviewed all patients who underwent VFG following tumor resection in Denmark between April 2010 and January 2022. Patients were treated at 1 of the 2 Danish sarcoma centers, either Aarhus University Hospital (AUH) or Copenhagen University Hospital (Rigshospitalet). All patients had tumors located in the appendicular skeleton. Follow-up duration was determined from the day of primary surgery until February 2024. Patients included in this study met the criterion of a minimum follow-up duration of 24 months unless mortality intervened before this specified period. No patients were lost to follow-up. Patient identification was facilitated through the Danish Sarcoma Database (DSD), a nationwide Danish database established in 2009. Data extracted from the DSD encompassed age, sex, tumor size, and location, histological type and grade, tumor necrosis, TNM stage (according to the American Joint Committee on Cancer), surgical margin (according to the Enneking classification), and administration of chemotherapy and/or radiation therapy. Patients’ medical records provided information on resection size, graft length, fixation technique, complications, relapse, and the patient’s vital status. The study was reported according to STROBE guidelines.

### Surgical technique

Preoperatively, all patients underwent magnetic resonance imaging (MRI) scans to facilitate surgical resection with safe margins. All tumors were surgically removed by en-bloc resection. Additional preoperative evaluation included CT angiography to establish the anatomy of the blood supply to the fibula graft. Cefuroxime was administered intravenously at the commencement of surgery and subsequently every 6 hours. This antibiotic regimen was maintained for at least 3 days following the surgical procedure. The fibula graft was harvested through a lateral approach by plastic surgeons, who also performed the vascular anastomosis. As the fibula graft was harvested, orthopedic oncologists conducted tumor resection concurrently. All primary surgeries were conducted as one stage. Osteosynthesis was achieved through internal fixation in all cases. There were 2 cases of reconstruction using the Capanna technique, in which the vascularized fibula graft was combined with a massive allograft. Both instances involved reconstruction after resection of the calcaneus. A double-barrel fibula flap was utilized in 5 cases of reconstruction, all involving the femur or tibia. This technique was chosen to address the cone-shaped anatomy created by the transition from the narrow diaphysis to the wide epiphysis and accommodate the significant forces transmitted through the lower extremity. In cases of reconstruction of the distal fibula, the contralateral proximal fibula was used. Weight-bearing was initiated when signs of graft union were affirmed radiographically.

### Chemo- and radiation therapy

Patients with Ewing sarcoma were treated according to the EuroEwing 99 and EuroEwing 2012 protocols, while patients with osteosarcoma were treated according to the Euramos protocol. Based on the tumor type, patients received chemotherapy both pre- and postoperatively. A good response to chemotherapy was defined as more than 90% tumor necrosis. Patients with Ewing sarcoma who did not show a good response to chemotherapy were treated with adjuvant radiotherapy.

### Radiographic outcome

Postoperatively, patients received regular outpatient follow-ups. Radiographs from these visits were evaluated for graft-specific outcomes. Graft union was attained when 1 of the following 3 criteria was met: (I) presence of an external bridging callus on 3 or more cortices on anteroposterior and lateral radiographs between the fibula graft and the recipient bone, (II) elimination of osteotomy lines on anteroposterior and lateral radiographs between the fibula graft and the recipient bone, (III) fusion in anteroposterior and lateral radiographs between the fibula graft and the adjacent bone or between the fibula graft–allograft construct and the joint. All had to meet the criteria if there were 2 or more junctions. Time to graft union was defined as the duration between the primary surgery and the attainment of graft union. Nonunion was defined as an absence of graft union 9 months postoperatively and no signs of healing for 3 months. Fibular graft hypertrophy was calculated using DeBoer and Wood’s method [20]. Significant fibular graft hypertrophy was defined as a more than 20% increase in diameter (Figure 4, see Appendix).

### Statistics

All graphs and statistical calculations were done in GraphPad Prism 10 (GraphPad Software, USA; https://www.graphpad.com/features). Continuous variables were reported as median and interquartile range (IQR). Categorical variables were reported as count. Continuous variables were compared using the Mann–Whitney U-test, for non-parametric unpaired data. Categorical variables were compared using Fisher’s exact test and reported as relative risk with a 95% confidence interval (CI). The Kaplan–Meier method was used to estimate time free of reoperation, limb salvage, overall survival, and disease-free survival. Calculations were performed using a level of significance of 0.05.

### Ethics, data sharing, funding, and disclosures

Ethical approval was received from the Danish National Center for Ethics (project number 2313297). Permission to access patients’ medical records was provided by the Central Denmark Region and the Capital Region of Denmark. The anonymized data supporting this study is available from the corresponding author upon reasonable request. Owing to privacy, the data is not included in this publication. This work was supported by the Danish Research Center for Cancer Surgery (ACROBATIC), the Danish Cancer Society (grant no. R317-A18255), and the Danish Comprehensive Cancer Center. Furthermore, the work was supported by the Child Cancer Foundation Grant (2022-8218) and the Danish Cancer Society (grant no. R352-A20505-B3708). The authors have no conflicts of interest to declare. Complete disclosure of interest forms according to ICMJE are available on the article page, doi: 10.2340/17453674.2025.42848

## Results

**Figure 1:**
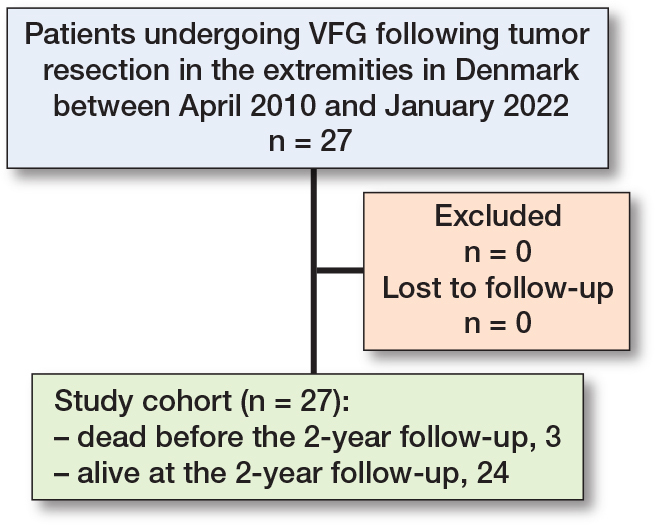
Patient flow diagram.

**Figure 2:**
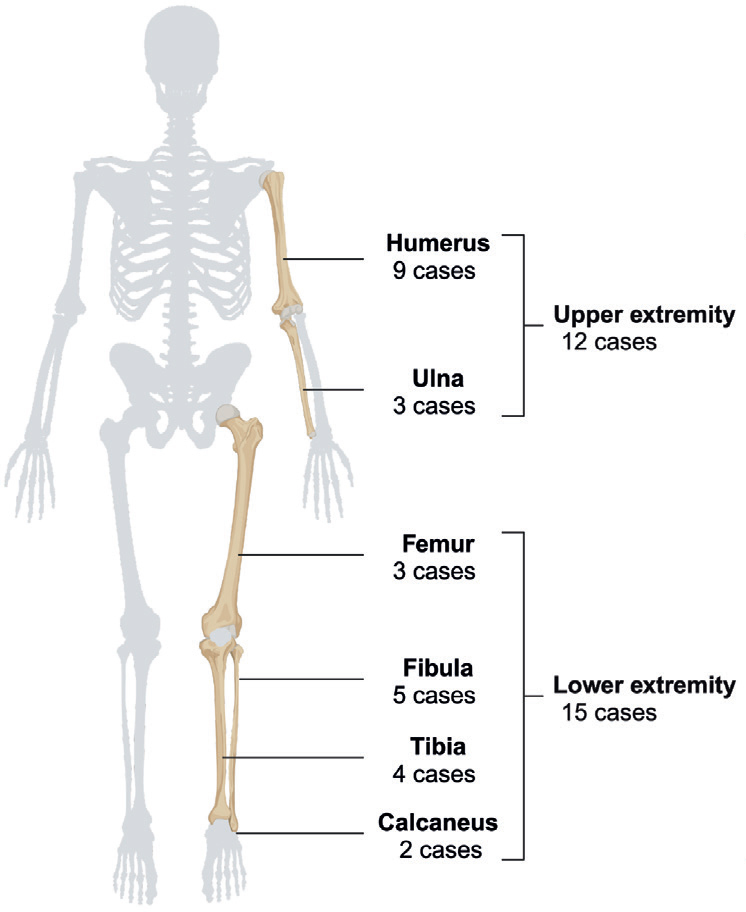
Distribution of the 27 primary tumors. Created in BioRender. Nielsen, C. (2025) https://BioRender.com/e89z429

The cohort comprised a total of 27 consecutive patients who underwent FVG following tumor resection in the extremities (Figure 1). There were 12 cases of upper extremity tumors and 15 cases of lower extremity tumors (Figure 2). The humerus was the most common location of primary tumors, with 9 cases. The median age at the time of primary surgery was 16 years (IQR 10–18) (Table 1).

**Table 1:** Patient characteristics (N = 27). Values are count or median (interquartile range)

Variable	Value
Female	11
Age	16 (10–18)
Pathology	
Ewing sarcoma	13
Osteosarcoma	12
Giant cell tumor	2
Tumor size, cm	6 (3–10)
Stage	
T1N0M0	16
T2N0M0	10
T2N0M1	1
Treated with	
chemotherapy	24
radiotherapy	4
Surgical margin	
Wide	23
Marginal	4
Resection length, cm	14 (10–17)
Graft length, cm	20 (15–22)

Chemotherapeutic treatment was administered to 24 patients in the cohort. Among these patients, 17 demonstrated a good response. 4 patients diagnosed with ES who exhibited a poor response to chemotherapy subsequently received adjuvant radiotherapy, with doses ranging from 45 to 54 Gray.

### Bone healing

Patients received follow-up for a median duration of 82 months (IQR 32–101) (Table 2).

**Table 2:** Follow-up data (N = 27). Values are count or median (interquartile range)

Variable	Value
Follow-up, months	82 (32–101)
Bone healing	
Primary graft union	17
Overall graft union	18
Months to union	13 (9–17)
Nonunion	6
Significant graft hypertrophy	8
Reoperations	20
Removal of osteosynthesis material	6
Wound revision	5
Nonunion revision	4
Limb-lengthening surgery	4
Osteosynthesis of fracture	3
Deep infection revision	2
Genu varum corrective surgery	2
Bleeding revision	1
Graft vein thrombosis revision	1
Hammer toe corrective surgery	1
Implant failure revision	1
Tendon reinsertion surgery	1
Graft fractures	10
Traumatic fracture	6
Stress fracture	5
Limb salvage	25
Survival	
2-year survival	24
5-year survival	22
Relapse	
Metastasis	8
Local recurrence	5
Months to metastasis	13 (3–14)
Months to local recurrence	14 (11–16)

17 patients (63%) attained primary graft union. Following secondary procedures, the overall rate of graft union increased to 18 patients in a median time of 13 months (IQR 9–17). Nonunion occurred in 6 reconstructions (a description of nonunion management is provided in Appendix).

### Complications and reoperations

**Figure 3:**
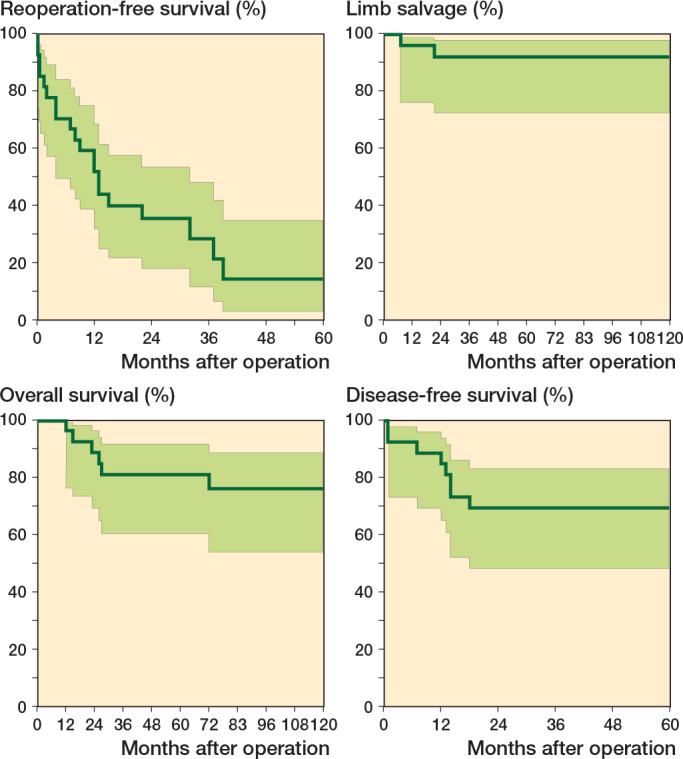
Kaplan–Meier survival curves following tumor resection and VFG (95% CI as light green area). Reoperation-free survival with reoperation for any complication in relation to the primary surgery being the endpoint (above, left). Limb salvage with amputation being the endpoint (above, right). Overall survival with death from any cause being the endpoint (below, left). Disease-free survival with relapse being the endpoint (below, right).

20 patients experienced complications necessitating surgical intervention. The proportion of patients free from any reoperation in relation to the primary surgery was 52% (CI 32–69) at 12 months postoperatively and 14% (CI 3–35) at 60 months (Figure 3). 6 patients underwent removal of osteosynthesis material after either attaining graft union or to create dynamization across the docking site. Wound revision procedures were performed in 5 of all primary surgeries, resulting in complete wound healing in all cases. 10 patients suffered a graft fracture during follow-up. Of these, 6 patients experienced a traumatic graft fracture, with 3 requiring open reduction and internal fixation due to fracture displacement. Additionally, 5 patients experienced a stress fracture, all of which were treated conservatively with a sling or a cast. A transient postoperative peroneal nerve paresis occurred following 7 primary surgeries, with all cases resolving spontaneously.

**Figure 4:**
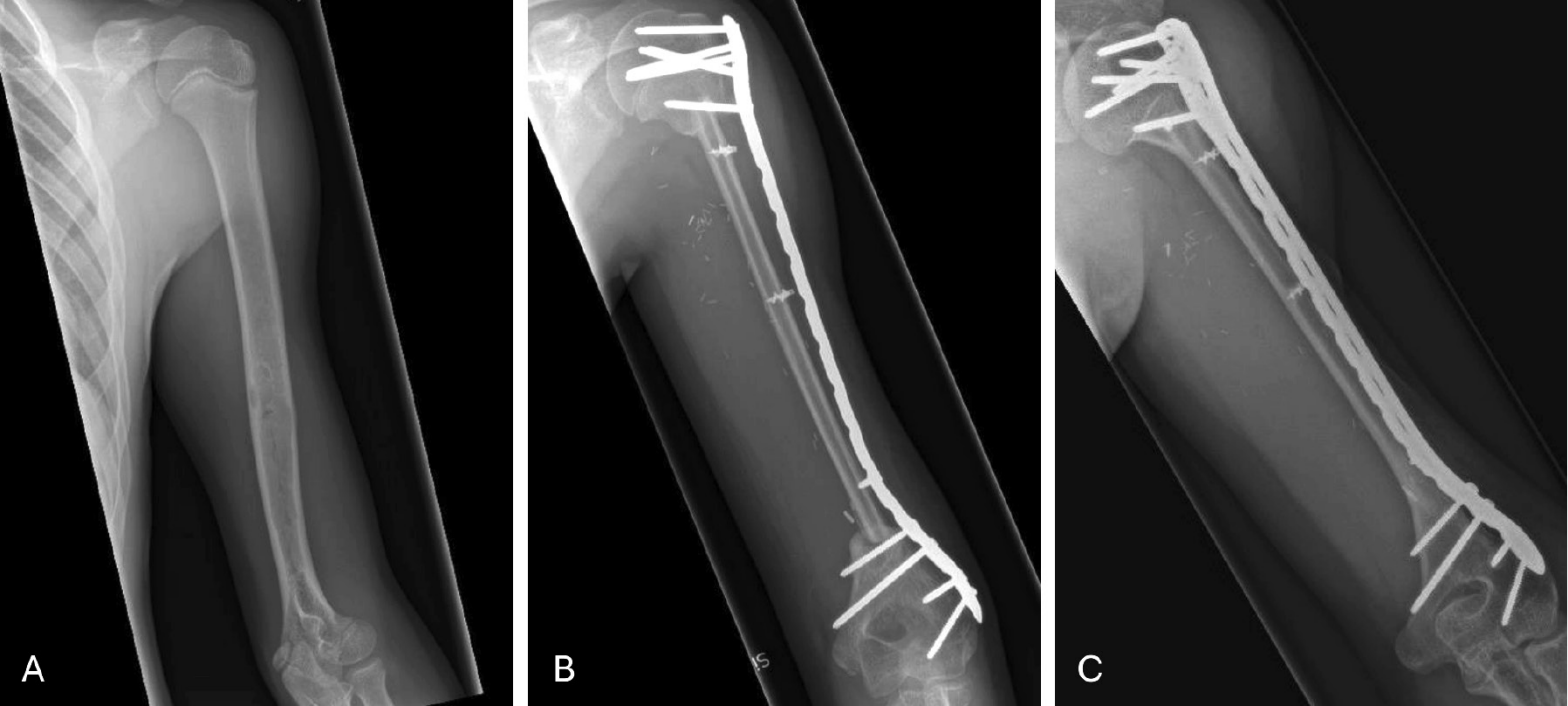
VFG following resection of osteosarcoma in the diaphysis of the humerus: (A) preoperative radiograph; (B) postoperative radiograph; (C) radiograph at 5 years postoperatively showing graft union and significant graft hypertrophy.

### Limb salvage and survival

The overall limb salvage rate was 93% (Figure 3). 2 patients in the cohort underwent above-the-knee amputations during the follow-up period. The first patient underwent amputation due to local recurrence 7 months after the primary surgery. The second patient underwent amputation 21 months postoperatively due to numerous complications resulting in impaired leg function.

Overall 2-year survival was 89% (CI 69–96) (Figure 3). 6 patients died, among whom 5 were directly attributable to disease relapse. The remaining patient died from leukemia 12 months after the primary surgery.

### Upper and lower extremity groups

Subgroup analysis based on primary tumor location into upper and lower extremity groups showed a bone defect size in the upper extremity group of 14 cm, which was significantly larger than bone defect size in the lower extremity group, 11 cm (P = 0.02) (Table 3). There was no difference between the 2 groups in the distribution of sex, age, pathology, tumor size, follow-up duration, and administration of chemotherapy or adjuvant radiotherapy.

**Table 3:** Comparison between upper and lower extremity groups

Variable	Upper extremity n = 12	Lower extremity n = 15	RR (CI)	P value
Bone healing				
Graft union	11	7	5.5 (1.3–31.5)	0.02
Nonunion	1	5	0.3 (0.06–1.2)	0.2
Significant graft hypertrophy	4	4	1.2 (0.5–2.6)	1.0
Reoperation				
One	7	13	0.5 (0.2–1.1)	0.2
Multiple	2	9	0.3 (0.8–0.9)	0.05
Graft fracture	7	3	2.4 (1.0–5.6)	0.06

RR = risk ratio; CI = 95% confidence interval.

Patients in the upper extremity group had a higher rate of union than those in the lower extremity group (Table 3). Although the median time to union was shorter in the upper extremity group compared with the lower extremity group there was no significant difference (11 months vs 15 months, P = 0.2). Furthermore, patients in the upper extremity group were less likely to undergo multiple reoperations than patients in the lower extremity group.

### Patients with OS or ES

There were significantly more females in the osteosarcoma group than in the Ewing sarcoma group (67% vs 15%, P = 0.02) (Table 4). However, no significant differences were observed between the 2 groups regarding age distribution, tumor location, tumor size, bone defect size, follow-up duration, or the administration of chemotherapy or adjuvant radiotherapy. There was no significant increase in the risk of impaired bone healing, reoperation, or fracture between the 2 groups.

**Table 4:** Comparison between patients with osteosarcoma and Ewing sarcoma

Variable	Osteo-sarcoma n = 12	Ewing sarcoma n = 13	RR (CI)	P value
Bone healing				
Graft union	10	8	1.9 (0.7–7.1)	0.4
Nonunion	2	4	0.6 (0.8–1.6)	0.7
Significant graft hypertrophy	4	4	1.1 (0.4–2.3)	1.0
Reoperation				
One	9	11	0.8 (0.4–2.1)	0.7
Multiple	3	8	0.4 (0.1–1.1)	0.1
Fracture	5	5	1.1 (0.4–2.4)	1.0

RR = risk ratio; CI = 95% confidence interval.

## Discussion

We aimed to evaluate (I) bone healing, (II) complications and reoperations, (III) limb salvage, and (IV) survival in a national cohort of 27 patients who underwent VFG following tumor resection in Denmark between 2010 and 2022. We found:

(I) 18 patients (67%) attaining graft union in a median time of 13 months (IQR: 9–17).
(II) 20 patients (74%) experienced complications during follow-up, necessitating surgical intervention.
(III) The overall limb salvage rate was 93%.
(IV) The 5-year overall survival rate was 81% (CI 61–92).

### Bone healing, complications, and reoperations

Our reported rate of primary union of 63% is lower compared with previous studies, reporting rates of 77–78% (16-18). After secondary procedures, our overall union rate was 67%, still below the previously reported overall union rates of 90–100% [16-18]. We observed a median time to union of 13 months, which is longer than the previously reported durations of 8–9 months [17,18]. However, our reported rate of nonunion of 22% aligns with previous reports of 12–23% [16-18], suggesting that our lower graft union rate was not solely attributable to a higher rate of nonunion compared with the previous studies.

Eward et al. reported an overall union rate of 100% and a 2-year survival rate of 100% in 30 patients treated with VFG following tumor resection in the extremities [17]. Conversely, we report a lower 2-year survival rate of 89%. Notably, 2 patients in our cohort died before achieving bone union, potentially contributing to our lower union rate. Chen et al. described an overall union rate of 93% in 25 patients treated with VFG after resection of extremity sarcoma and reported no cases of amputation [16]. In contrast, the amputation rate was 7% in our cohort. Both patients who underwent amputation in our study had not attained bone union, which may have contributed to our lower union rate. Hsu et al. reported an overall union rate of 90% in 30 patients treated with VFG following tumor resection in the extremities [18]. Their study included 23 patients with malignant tumors and 7 with benign tumors, whereas our study comprised 25 patients with malignant diagnoses and 2 patients with a benign diagnosis. The higher proportion of malignant tumors in our study likely led to a greater number of patients receiving chemotherapy and radiation therapy. Multiple studies have shown that chemotherapy and/or radiation therapy can impair bone healing in reconstructive surgery using bone grafts [21,22]. Thus, our lower overall graft union rate and prolonged time to union may be attributable to several contributing factors such as our reported rate of overall 2-year survival, amputations, and proportion of patients undergoing chemo- and radiation therapy.

Patients in the upper extremity group demonstrated a significantly higher rate of graft union and were less likely to undergo multiple reoperations than those in the lower extremity group in accordance with Hilven et al. [23]. Xu et al. also found higher complication rates in lower extremity reconstructions, although the difference was not statistically significant [24]. An inherent anatomical disparity exists between the upper and lower extremities, as the humerus has a smaller diameter than the femur. Therefore, the diameter of the humerus more closely resembles that of the fibular graft. Additionally, the weight-bearing bones of the lower extremity are subjected to significantly higher mechanical loads than those of the upper extremity. The combination of anatomical and biomechanical factors, along with the more intricate surgical techniques required for lower extremity reconstructions, may explain their lower graft union rates compared with upper extremity reconstructions. Furthermore, Hilven et al. reported a higher complication rate in pediatric patients compared with adults [23]. In our cohort, no increased risk of impaired bone healing or complications was observed between patients over and under 16 years of age (data not presented).

Hariri et al. reported a higher union rate in patients with Ewing sarcoma compared with osteosarcoma, hypothesizing that methotrexate used in osteosarcoma treatment inhibits bone formation [25]. We found no significant difference in bone healing or complication rates between patients with OS or ES.

### Limb salvage and survival

We reported a limb salvage rate of 93%, which is consistent with previously reported rates of 93–100% [16-18,23]. During the follow-up period, 3 grafts (11%) were removed due to local recurrence, nonunion, or infection, similar to the 10% removal rate reported by Hsu et al. for the same reasons [18]. Our overall 2-year survival rate of 89% is comparable to the rates reported by previous studies of 80-100% [16-18].

### Strengths

Our study comprises a national cohort of all patients treated in Denmark between 2010 and 2022, with no patients lost to follow-up. Our study boasts a substantial median and minimum follow-up duration, allowing for long-term evaluation of the patients. Patients treated at both Danish sarcoma centers were included, meaning that multiple specialized surgical teams conducted the surgeries. The execution of all surgeries as 1-stage procedures contributed to uniformity.

### Limitations

The study’s findings were constrained by the inherent biases associated with a retrospective study design. Moreover, the study’s statistical power to detect outcome differences was reduced due to the rarity of BS and the relatively uncommon nature of the procedure.

### Conclusion

VFG following tumor resection was associated with a graft union rate of 67%, a high frequency of reoperations, a high limb salvage rate (93%), and a 5-year survival rate of 81%.

*In perspective,* VFG remains a valuable option in limb salvage surgery with acceptable long-term outcomes.

## Figures and Tables

**Figure 5:**
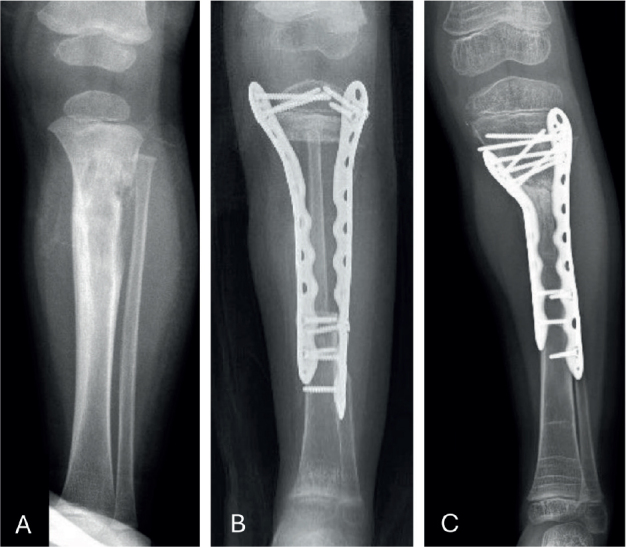
VFG following resection of Ewing sarcoma in the metadiaphysis of the tibia: (A) preoperative radiograph; (B) postoperative radiograph; (C) radiograph at 32 months postoperatively showing a persistent nonunion at the proximal graft–host junction.

## Notes

Contributions are consistent with the 4 criteria of ICMJE. Design of study: CLN, MMP, TBH. Data acquisition: CLN, DHD, PVG, MMB, MMP, TBH. Data analysis: CLN. Data interpretation: CLN, TBH. Paper draft: CLN. Critical review: CLN, DHD, PVG, LLH, PEW, BJK, MMB, MMP, TBH.

Handling co-editor: Ilkka Helenius

*Acta* thanks Aare Märtson, Klaus Dieter Parsch, and Panagiotis Tsagkozis for help with peer review of this manuscript.

## Appendix

### Nonunion management

The first patient in the cohort to develop a nonunion experienced a tibial nonunion and underwent revision surgery 32 months after the primary surgery, using autograft from the femur. However, graft union was not attained before the patient died of relapse. The second patient in the cohort to develop a nonunion was initially treated with a modified Capanna reconstruction of the calcaneus, where an adult femoral head allograft was combined with a pedicled vascularized fibula graft. The patient developed a nonunion in the arthrodesis between the vascularized fibula graft–allograft construct and the cuboid bone. Because the nonunion remained asymptomatic, it was left untreated. The third patient in the cohort to develop a nonunion in the humerus and underwent revision surgery 12, 22, and 28 months after the primary surgery using bone void filler and subsequent hyperbaric oxygen therapy. Despite these interventions, graft union was not attained and the patient received a custom-made prosthesis. The fourth patient in the cohort who developed a nonunion experienced a femoral nonunion, knee and ankle valgus, and a symptomatic leg length discrepancy. These factors collectively contributed to impaired leg function. Consequently, an above-knee amputation was performed 21 months after the primary surgery. The fifth patient in the cohort to develop a nonunion in the femur and underwent revision surgery using bone void filler 12 months after the primary surgery. Subsequently, bone healing was attained 29 months after the primary surgery. The sixth patient in the cohort who developed a nonunion experienced a tibial nonunion. Revision surgery was performed 26 months after the primary procedure using allograft chips. However, at the most recent radiographic evaluation, conducted 32 months postoperatively, graft union had not yet been attained (Figure 5).
